# An *in situ* image-based phenotyping system for hydroponic maize seedling roots based on DB-UNet and customized skeleton-based analysis

**DOI:** 10.3389/fpls.2026.1851523

**Published:** 2026-06-17

**Authors:** Yurong Guo, Yue Huang, Chenxu Zhu, Luxu Tian, Jingxuan Zhang, Yimeng Li, Yitong Liu, Hongbin Wu, Xiuqing Fu

**Affiliations:** 1 College of Engineering, Nanjing Agricultural University, Nanjing, China; 2 College of Smart Agriculture (College of Artificial Intelligence), Nanjing Agricultural University, Nanjing, China

**Keywords:** maize root, root phenotyping, DB-UNet, semantic segmentation, skeleton extraction, root length estimation, shoot-root correlation

## Abstract

Root phenotype is a key agronomic trait affecting maize growth and development. *In situ* observation and high-precision root phenotypic analysis provide important support for monitoring maize growth. Traditional root phenotyping methods lack *in situ* monitoring capabilities, and existing models have limited accuracy in root segmentation. To address these issues, we developed a crop root phenotyping system integrating crop cultivation and data collection. We also proposed a DB-UNet model for hydroponic maize root segmentation. DB-UNet builds a CNN-ViT dual-branch parallel structure during encoder downsampling level. The lightweight ViT branch uses sequential downsampling to achieve global topological dependency modeling while reducing computational costs. An attention fusion module dynamically calibrate dual-branch features weights, achieving complementary fusion of local root edge details and global context information. we constructed a mixed loss function combining Dice loss, Focal loss, and structural consistency KL loss to solve class imbalance, hard sample segmentation, and semantic divergence of dual-branch features. On our custom hydroponic maize root dataset, DB-UNet achieved an mIoU of 91.02%, an FG IoU of 82.78%, and a Centerline-Dice of 97.72%.Compared to classic UNet, mIoU, FG IoU, and Centerline-Dice increased by 0.92%, 1.84%, and 1.99%, respectively. Plant-level five-fold cross-validation further showed that DB-UNet maintained stable segmentation performance across different plant-level partitions. Based on DB-UNet segmentation results, we propose a custom skeleton-based algorithm for multi-trait root phenotyping, enabling the extraction of total root length and root branch points. Root area is calculated from binary mask pixel statistics. Compared to the traditional Zhang-Suen algorithm, the average relative error of root length measurement is reduced to 3.14%, which is 8.42 percentage points lower than the traditional method. Furthermore, we analyzed relationships between segmentation accuracy metrics and phenotypic relative errors. Higher segmentation quality generally led to lower phenotypic relative errors and more reliable trait measurements. In particular, Centerline-Dice was closely associated with root length estimation, whereas pixel-level segmentation consistency was more closely related to root area measurement. Pearson and Spearman correlation analyses showed a strong positive correlation between maize plant height and total root length, with coefficients of 0.8466 and 0.8634, respectively.

## 1 Introduction

The root system is a critical underground organ of crop plants, playing a central role in anchorage as well as the uptake and transport of water and mineral nutrients (Bucksch et al., 2014). Root phenotypic traits largely reflect the adaptability of crops to environmental conditions and their growth status. Therefore, systematic investigation of root phenotypes is considered a key strategy for improving resource-use efficiency and advancing the second “Green Revolution” (Li et al., 2022). Accurate and quantitative monitoring of dynamic root phenotypic changes provides essential data support for the identification of superior traits, variety screening, and genetic breeding, thereby enhancing breeding efficiency and accelerating crop improvement.

Maize (Zea mays L.) is one of the most widely cultivated and productive cereal crops worldwide, serving as a major source of starch for both human consumption and livestock feed (Hochholdinger and Tuberosa, 2009). In the context of rapid global population growth, increasing pressure on arable land resources, and intensified climate change, improving maize yield and stability has become a critical agricultural challenge (Lu et al., 2020).As the primary organ for water and nutrient uptake, the maize root system—particularly its architecture and spatial distribution—is regarded as the “underground foundation” determining yield potential, yield stability, and stress resistance (Shao et al., 2021). Therefore, efficient acquisition and accurate analysis of maize root phenotypes are of great scientific significance and hold broad application prospects for addressing current agricultural challenges.

Traditional root monitoring methods mainly include excavation, soil coring, and minirhizotron techniques, each with distinct advantages and limitations. The excavation method is one of the earliest and most widely used approaches, in which intact root systems are obtained by digging soil for subsequent measurement. While this method provides comprehensive information on root morphology and biomass, it is highly destructive and unsuitable for continuous dynamic monitoring. The soil coring method estimates root density or root length density by analyzing soil samples. Although it reduces disturbance to some extent, it still suffers from limited sample representativeness and weak *in situ* monitoring capability. In addition, the minirhizotron technique enables non-destructive observation of roots using transparent tubes combined with imaging devices. However, its effectiveness is constrained by the spatial distribution of roots and the limited field of view, resulting in restricted accuracy for dynamic monitoring (Zheng et al., 2024).To overcome the limitations of traditional methods, particularly their destructiveness and limited *in situ* observation capability, transparent rhizobox systems have been developed in recent years for non-destructive monitoring of maize seedling roots (Nagel et al., 2012; Atkinson et al., 2019). These systems are typically constructed from transparent materials, allowing direct visualization of root growth trajectories under hydroponic conditions. Hydroponic cultivation provides a controlled environment for water and nutrient supply, enabling more predictable root growth patterns and facilitating high-quality imaging, while avoiding measurement errors caused by soil disturbance. The advantages of this approach are threefold. First, it enables dynamic and continuous monitoring of root growth. Second, the combination of transparent rhizobox systems and hydroponic conditions improves image clarity and root resolution, which is beneficial for subsequent automated image analysis. Third, this method is non-destructive and highly reproducible, making it suitable for high-throughput screening and phenotypic analysis of multiple maize plants, thereby providing reliable data support for breeding and root growth mechanism studies (Baiyin et al., 2021).

Deep learning techniques have promoted plant root phenotyping toward high-throughput, automated, and high-precision analysis. Compared with traditional image processing and shallow machine learning methods, semantic segmentation models can learn discriminative features in an end-to-end manner and have become an important approach for root extraction and subsequent phenotypic quantification. Representative architectures, including U-Net, U-Net++, DeepLabv3+, SegFormer, and other Transformer-based models, have been widely explored for root image segmentation and related plant phenotyping tasks. U-Net adopts an encoder–decoder architecture with skip connections, enabling the fusion of high-level semantic information and low-level spatial details, and has shown stable performance in both biomedical image segmentation and plant root segmentation tasks (Ronneberger et al., 2015). DeepLabv3+ introduces atrous spatial pyramid pooling to enlarge the receptive field and improve multi-scale feature representation (Chen et al., 2018). SegFormer employs a Transformer-based encoder to enhance long-range dependency modeling and has demonstrated strong performance in semantic segmentation tasks (Xie et al., 2021). In root phenotyping studies, Smith et al. applied U-Net-based models to root segmentation in soil images, demonstrating their robustness under complex background conditions (Smith et al., 2020). More recently, hybrid architectures combining convolutional neural networks and Transformers have been introduced to further improve segmentation performance. These models aim to combine the local feature extraction ability of CNNs with the global dependency modeling capability of Transformers, which is particularly relevant for root images containing thin, continuous, and highly branched structures. Overall, deep learning-based segmentation methods provide important technical support for identifying primary roots, lateral roots, and root structural continuity, thereby facilitating the quantitative extraction of phenotypic traits such as root length, root projected area, and branching-related traits. However, fine-root segmentation, topology preservation, and stable downstream trait extraction remain challenging for hydroponic maize root images due to sparse foreground pixels, weak boundaries, and background interference.

With the development of plant phenomics, root analysis has gradually evolved from a single image segmentation task into a complete analytical workflow involving structural reconstruction, trait extraction, and standardized representation. In recent years, various root phenotyping tools and platforms have been proposed. For example, RootNav 2.0 combines deep learning-based segmentation with path-searching algorithms to achieve root system architecture (RSA) reconstruction and RSML output (Yasrab et al., 2019). RhizoVision Explorer provides standardized root trait extraction functions and can quantify multidimensional phenotypic traits such as root length, root area, and branching structure (Seethepalli et al., 2021). RootEx 2.0 and related methods integrate image segmentation, keypoint detection, and graph-based reconstruction to establish end-to-end root phenotyping pipelines (Dadi et al., 2025a). In addition, interactive annotation tools such as RootTracer provide important support for the construction of high-quality ground-truth data (Dadi et al., 2025b). These studies indicate that current root phenotyping analysis is shifting from pixel-level segmentation toward structural-level understanding and multi-trait characterization. However, in the context of maize seedling root analysis, existing methods still face challenges in fine-root segmentation accuracy, topological structure preservation, and high-throughput automated analysis due to the slender morphology, dense overlap, and low contrast of root systems.

In summary, existing root observation systems still exhibit limitations in terms of continuous *in situ* monitoring capability and non-destructive measurement. In addition, current image segmentation models still have room for improvement in accurately capturing fine roots and small targets. To address these challenges, this study employs a transparent rhizobox system for *in situ* imaging and proposes a DB-UNet model, developed based on the U-Net architecture, for segmenting hydroponically grown maize roots. Furthermore, a root length estimation algorithm is constructed based on the segmentation results to achieve efficient and accurate quantification of root phenotypic traits. Building upon these methods, a correlation analysis between aboveground plant height and belowground root length is also conducted. The main contributions of this study are summarized as follows:

### 1.1 Dataset construction

A hydroponic maize root imaging dataset based on a transparent rhizobox system was established. Root images were acquired *in situ* and manually annotated, and subsequently divided into training, validation, and test sets according to the experimental design, providing a reliable data foundation for model training and performance evaluation.

### 1.2 DB-UNet model based on U-Net improvement

A task-specific DB-UNet segmentation model was developed for hydroponic maize roots, which are characterized by thin structures, sparse foreground pixels, low contrast, and strong topological continuity. Rather than simply stacking CNN and Transformer modules, DB-UNet establishes a cooperative modeling mechanism between local detail extraction and global structural perception within the U-Net encoder. The CNN branch enhances local representations of root edges, root tips, and fine lateral roots, while the lightweight ViT branch models long-range dependencies through a sequence downsampling strategy to improve primary-root continuity and global structural awareness. In addition, an attention fusion module was designed to alleviate semantic conflicts caused by direct concatenation of heterogeneous CNN and ViT features. A hybrid loss function was further introduced to jointly constrain region overlap, hard samples, and inter-branch structural consistency. This design makes the model more suitable for fine-root segmentation and topology preservation in hydroponic maize root images.

### 1.3 Multi-trait root phenotyping method and validation based on segmentation results

Based on the DB-UNet segmentation results, we developed a customized skeleton-based analysis method for extracting representative root traits, including total root length, projected root area, and branch point count. The proposed method combines preprocessing, skeleton extraction, burr pruning, and improved chain-code statistics to improve the stability of root length estimation. These traits provide useful morphological descriptors of hydroponic maize seedling roots. Furthermore, based on the extracted phenotypic traits, we further investigated the impact of segmentation quality on downstream phenotypic trait extraction.

### 1.4 Correlation analysis between aboveground and belowground traits

Based on the root phenotyping system, the DB-UNet segmentation model, and the proposed root length extraction algorithm, a correlation analysis between plant height and root length in maize seedlings was conducted. Heatmap analysis reveals a significant and strong positive correlation between these two traits.

## 2 Materials and methods

### 2.1 Experiment equipment

**Figure 1:**
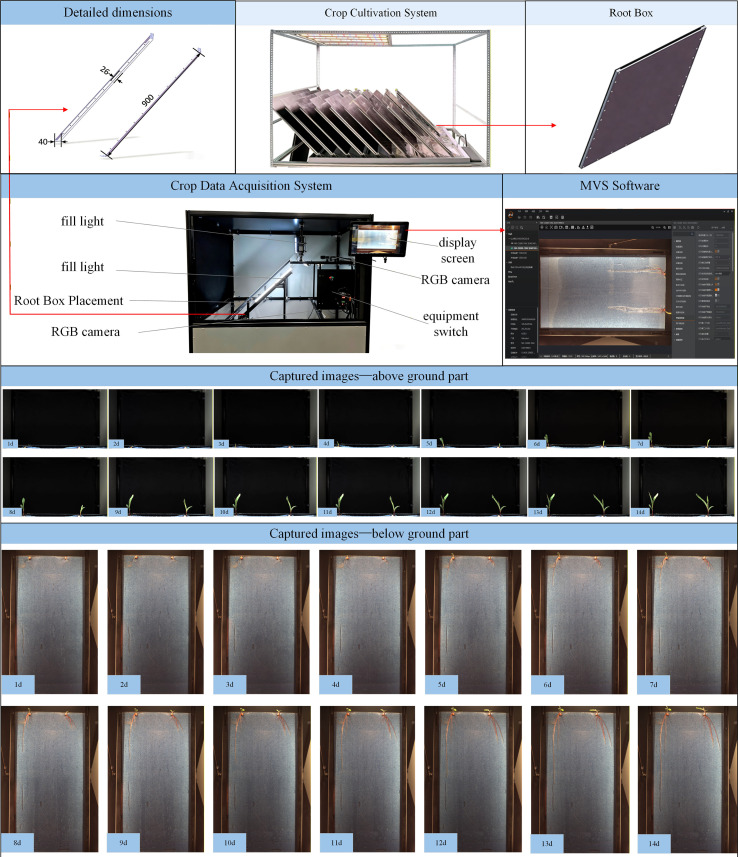
Crop root phenotyping system.

The crop root phenotyping system used in this study consists of two main modules: a crop cultivation system and a data acquisition system, as shown in **Figure 1**.

The crop cultivation system is mainly constructed with structural profiles and angle steel. Its overall dimensions are 2305 × 1035 × 1840 mm (length × width × height). A row of grow lights is installed at the top of the system to provide light for crop growth. It uses a modular design and consists of 20 flat root boxes. Each root box measures 800 × 410 × 5 mm (width × length × height) and features a double-layer structure. One side is transparent to allow camera imaging of the roots. The other side is black to block light. These two plates are secured with 27 M3 × 10 stainless steel screws (seven at the bottom and ten on each side). The support frame holds the root boxes at a 45° angle. This design guides the roots to grow downward and keeps them as close to the transparent side as possible.

The data acquisition system is also constructed with a profile frame. Its inner surface is covered with blackout cloth to ensure a stable imaging environment. The front of the device features sliding doors, which are completely closed during imaging to block external light interference. The root box placement area is also tilted at 45°. Inside the device, four light sources are installed: two on the sides of the root box and two at the top. This setup ensures uniform lighting in the imaging area. The core components of the data acquisition system are two Hikvision CS20010GC industrial RGB cameras and the MVS image acquisition software. These two cameras capture images of the aboveground and belowground plant parts, respectively. They are equipped with Sony IMX183 sensors, offering a high resolution of 5472 × 3648 pixels and a pixel size of 2.4 μm. They use a GigE interface for high-speed data transmission.

### 2.2 Data acquisition and preprocessing

**Figure 2:**
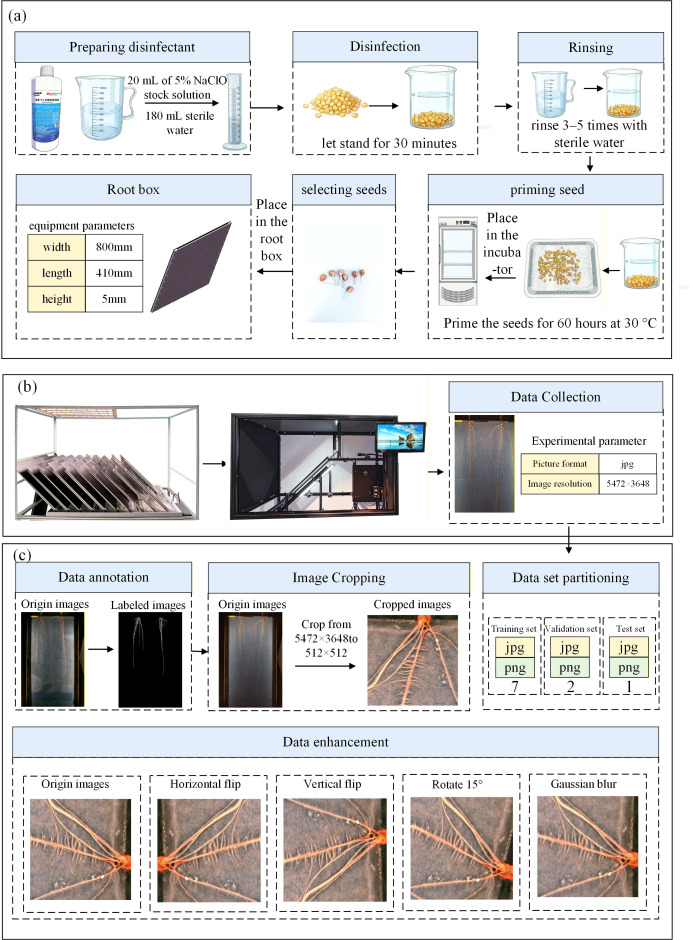
**(a)** Introduction process **(b)** Data collection process **(c)** Dataset construction process.

We selected 22 uniform and plump maize seeds for the hydroponic experiment. The seed cultivation process is shown in **Figure 2a**. We used the crop cultivation system and the data acquisition system to collect the root data of the hydroponic maize. The experiment lasted for two weeks. Images were captured once a day, resulting in a total of 154 images. The dataset acquisition workflow is shown in **Figure 2b**. During the first four days, the maize roots were very small. Therefore, we removed the 44 images collected during this period, as they provided little help for model training. The dataset was constructed using the workflow shown in **Figure 2c**. The original captured images have a resolution of 5472 × 3648. This high resolution preserves the spatial information of fine structures, such as root tips and lateral roots. Afterward, we used Adobe Photoshop to annotate the maize roots. The annotation followed a binary classification rule. The maize root regions were set as the foreground with a pixel value of 1. Non-target regions, such as the hydroponic background, were set as the background with a pixel value of 0. The label images were saved in PNG format. To avoid data leakage caused by highly similar images from the same plant, the dataset was divided at the plant level. Specifically, the 10-day continuous growth data of 22 maize seedlings were randomly divided into training, validation, and test sets at an approximate ratio of 7:2:1. All original images, cropped image patches, and corresponding annotation masks derived from the same maize seedling were kept in the same subset. Before training, all original JPG images and PNG labels were cropped to a uniform resolution of 512 × 512. Images without roots were then removed. After cropping, there were a total of 1547images. This formed the final dataset used for model training.Maize roots have a slender structure and specific growth characteristics. Excessive data augmentation can easily cause structural distortion. Therefore, we designed a mild geometric augmentation strategy. This approach increases the diversity of the dataset without destroying the topological structure of the roots. The augmentation process relied solely on geometric transformations. We did not apply perturbations to brightness, contrast, or color. This ensured that the true distribution of the root images was maintained. Specifically, we randomly applied horizontal flipping, vertical flipping, rotation within ±15°, minor translation of no more than 5%, and Gaussian blur. The execution probabilities for these operations were 50%, 50%, 30%, 20%, and 10%, respectively.

### 2.3 Construction of maize root semantic segmentation model based on DB-UNet

U-Net (Ronneberger et al., 2015) is a semantic segmentation model based on Convolutional Neural Networks (CNN). Originally designed for medical image segmentation, it is now widely used in various fields such as agriculture, medicine, and industry due to its concise architecture and superior performance. The model uses an encoder-decoder framework. In the encoding stage, convolutional and pooling layers progressively extract deep semantic features to downsample the image. In the decoding stage, deconvolutional layers restore the spatial resolution. Meanwhile, skip connections fuse shallow texture features from the encoding stages with deep semantic features. This effectively compensates for the loss of spatial information during decoding and improves the accuracy of segmentation boundaries.

**Figure 3:**
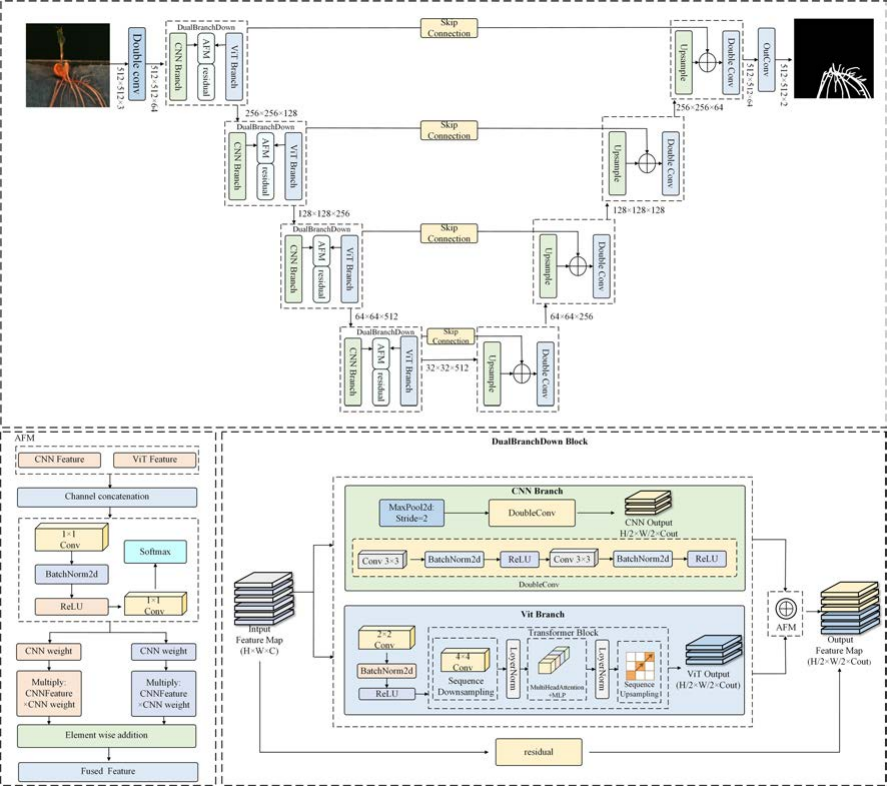
Network architecture of DB-UNet.

However, convolutional operations are inherently local. Although pooling layers expand the receptive field, the loss of spatial resolution in deep networks makes it difficult for the model to distinguish fine root tips from background noise. Furthermore, CNN struggles to establish long-range dependencies between pixels. For targets with continuous and slender structures like roots, this often leads to interrupted segmentation results or fractured roots. Additionally, U-Net simply concatenates features in the skip connections, ignoring the distributional differences between deep semantic and shallow texture features. This results in rigid feature fusion and limits segmentation performance. In contrast, the Transformer architecture uses a self-attention mechanism to effectively model long-range dependencies, overcoming the local receptive field limitations of CNN. As a classic application of Transformers in computer vision, Vision Transformer (ViT) (Dosovitskiy et al., 2021) can efficiently extract global contextual information. And hydroponic maize root segmentation differs from general natural-image segmentation tasks. The main challenge is not only foreground–background classification, but also the preservation of the continuity of thin root structures. Root tips and lateral roots occupy only a small number of pixels and can easily disappear after repeated downsampling. Meanwhile, the primary root and lateral roots exhibit strong spatial continuity, and relying only on local convolutional features may lead to fragmented root predictions. Therefore, the design objective of DB-UNet was not to simply increase architectural complexity, but to improve local detail recognition and global topological perception partially. Based on this motivation, we improved the U-Net model and proposed the DB-UNet, as shown in **Figure 3**. The specific improvements are as follows:

Introduction of a Lightweight ViT Branch (D-UNet): A dual-branch parallel structure is implemented at each downsampling level. The CNN branch captures complex edge details to ensure the sharpness of root boundaries and prevent fine structures from disappearing after multiple downsampling steps. Simultaneously, the Transformer branch establishes global topological constraints through self-attention. To solve the memory overflow issues of Transformers when processing high-resolution images, we introduced a sequence downsampling strategy. A built-in 4×4 stride convolution pre-compresses the token sequence, reducing the spatial dimensions to H/4×W/4and the total number of tokens N to 1/16 of the original. This significantly reduces computational costs while maintaining a global receptive field. Multi-head attention is then performed on these compressed tokens. This reduction in tokens allows the model to process 512×512 inputs without memory overflow. After computation, bilinear interpolation restores the feature maps to their original size for fusion with the CNN branch.

Introduction of the Attention Fusion Module (AFM)(DB-UNet): Features from the CNN branch primarily respond to gradient changes at root edges, while features from the ViT branch aggregate long-range dependencies and low-frequency structural information. Direct concatenation causes aliasing in the feature space and introduces non-constructive noise. To address this, we propose the Attention Fusion Module (AFM). The AFM learns a spatially adaptive weight to dynamically recalibrate the contributions of the two branches at the pixel level. By using spatial and channel attention mechanisms, the module adaptively assigns weights to the CNN and ViT feature maps. It assigns higher weights to CNN features in edge regions and higher weights to ViT features for primary root connectivity, achieving complementarity between local details and global macro-information.

Let the feature maps input to the fusion module be $F_{CNN}$ and $F_{ViT}$. First, we concatenate the features from the two heterogeneous branches along the channel dimension to construct a joint feature tensor $F_{\text{con}cat}$ containing complete information, as shown in Equation 1:

(1) $$F_{concat}=\text{Concat}(F_{CNN},F_{ViT})\in {\mathbb{R}}^{B\times 2C\times H\times W}$$

To capture the spatial complementarity between features, we use a parameterized mapping function $F_{attn}$ to generate attention weights. Specifically, a 1×1 convolutional layer compresses the channel dimension, and a Sigmoid activation function maps the output to the (0, 1) interval to form the spatial attention A, as shown in Equation 2:

(2) $$\text{A}=\sigma (W_{\theta }(F_{concat}))\in {\mathbb{R}}^{B\times 1\times H\times W}$$

where $W_{\theta }$ represents the weight parameters of the convolutional operation.

Finally, we use a complementary weighting strategy to fuse the features based on the generated attention. To prevent gradient vanishing and preserve low-level information from the original input, we introduce a residual connection. The final fused feature $F_{fused}$ is defined in Equation 3:

(3) $$F_{used}=\underset{\text{Local Focus}}{\underset{︸}{\left(\text{A}⊙F_{cnn}\right)}}+\underset{\text{Global Context}}{\underset{︸}{(1-\text{A})⊙F_{vit}}}+R(X_{in})$$

where ⊙denotes the hadamard product, 1is an all-ones matrix, and $R(X_{in})$ is the residual mapping from the previous layer’s input.

Introduction of the Mixed Loss Function: In the phenotyping task of hydroponic maize roots, the images show a significant foreground-background imbalance. Moreover, the two-branch structure may cause semantic divergence in feature distribution during the early training stage. If relying solely on supervised loss, the two branches might fit different feature subsets, leading to uncertainty in the fused predictions. To address these issues, this paper designs a mixed loss function, SL Loss, which combines Dice Loss, Focal Loss, and a structural consistency loss (KL divergence).

#### 2.3.1 Dice Loss

Due to the sparse distribution of root systems, standard pixel-level cross-entropy loss is often dominated by massive background pixels. This causes the model to predict an all-background image to reach a local minimum. Therefore, this paper uses Dice Loss (Milletari et al., 2016) as the main supervision signal. It aims to directly optimize the set overlap between the predicted root skeleton and the ground truth mask. Dice Loss is invariant to changes in foreground scale, which effectively relieves the class imbalance problem. Its expression is shown in Equation 4:

(4) $$L_{dice}=1-\frac{2\sum_{i=1}^{N}\;p_{i}g_{i}+\epsilon }{\sum_{i=1}^{N}\;p_{i}+\sum_{i=1}^{N}\;g_{i}+\epsilon }$$

Where N is the total number of pixels in the image; $p_{i}\in [0,1]$ represents the predicted probability that the i pixel belongs to the maize root; $g_{i}\in \{0,1\}$ represents the ground truth label of the i pixel; is a smoothing term used to prevent a zero denominator and stabilize the training process.

#### 2.3.2 Focal Loss

To address the lack of targeted focus in Dice Loss, this paper introduces Focal Loss (Lin et al., 2017). By introducing a focusing parameter, Focal Loss reduces the weight of large black background areas. This forces the model to focus on hard-to-classify pixels, such as root tips and overlapping points. Its expression is shown in Equation 5. After introducing Focal Loss, the model’s ability to capture fine root structures is significantly enhanced.

(5) $$L_{focal}=-\frac{1}{N}\sum_{i=1}^{N}\alpha {\left(1-p_{t,i}\right)}^{\gamma }\log(p_{t,i})$$

#### 2.3.3 Structural consistency loss

The CNN branch focuses on local details, while the ViT branch focuses on global dependencies. Consequently, they may produce semantic divergence in feature distribution during early training. If relying only on the supervised losses mentioned above, the two branches might fit different feature subsets, causing uncertainty in the fused predictions. To regularize the latent space, we use symmetric KL divergence as a regularization term. This forces the output probability distributions of the two branches to approach each other to achieve mutual learning, as shown in Equation 6:

(6) $$L_{KL}=\frac{1}{2}(D_{KL}(P_{cnn}∥P_{vit})+D_{KL}(P_{vit}∥P_{cnn}))$$

Where $P_{CNN}$ and $P_{ViT}$ are the Softmax output distributions of the two branches, respectively. This loss term does not introduce new external labels. Instead, it improves the robustness of the model against noise through a self-distillation mechanism between branches.

#### 2.3.4 Overall Mixed Loss Function

In summary, to balance region overlap, hard and easy sample mining, and feature consistency, we apply a weighted combination of the three losses above. This builds the final composite optimization objective, as shown in Equation 7:

(7) $$L_{SL}=\lambda_{1}L_{focal}+\lambda_{2}L_{dice}+\lambda_{3}L_{KL}$$

Where a weight of $\lambda_{1}=0.4$ is used to enhance detail classification, a weight of $\lambda_{2}=0.5$ is used to ensure the completeness of the overall skeleton, and a weight of $\lambda_{3}=0.1$ serves as an auxiliary regularization term.

### 2.4 Model training parameters and evaluation metrics

The experiment was conducted on a Linux operating system. The hardware included an Intel(R) Xeon(R) Platinum 8474C processor (15 cores) and 80 GB of memory. The GPU was an NVIDIA GeForce RTX 4090D with 24 GB of video memory, and the GPU driver version was 580.105.08. Python 3.8 was used as the development language. The deep learning framework was PyTorch 2.0.0, with a selected CUDA version. The model training parameters are shown in **Table 1**.

**Table 1:** Model training parameters.

Parameter	Value
Total Epochs	200(30 freeze epochs for ViT)
Batch Size	8
Image Size	512×512
Optimizer	AdamW
Initial LR(CNN Branch)	0.0001
Initial LR(ViT Branch)	5×10^-6^
Weight Decay	0.0005
Workers	4
Mixes Precision Training	FP16

To objectively and comprehensively evaluate the model’s performance in the hydroponic maize root segmentation task, this study used several metrics to evaluate segmentation accuracy. These included mean Intersection over Union (mIoU), Foreground Intersection over Union (FG IoU), Recall, and the Dice similarity coefficient. Centerline-Dice was introduced as topology-aware metrics to assess the model’s ability to preserve root centerline continuity, skeleton integrity, and branching structures.

mIoU measures the overlap between the segmentation results and the ground truth. FG IoU calculates the overlap between the segmentation results and the foreground root regions in the ground truth. Recall measures the model’s ability to cover the true foreground root regions. The Dice similarity coefficient effectively amplifies segmentation errors in small target areas. This more accurately reflects the model’s ability to segment fine root details. Centerline-Dice is used to evaluate the preservation of root centerline continuity and skeleton-level structural integrity. The specific calculation formulas are shown in Equations 8-14.

(8) $$mIoU=\frac{1}{1+1}\sum_{i=0}^{1}\frac{TP}{TP+FN+FP}$$

(9) $$\text{FG IoU}=\frac{TP}{TP+FN+FP}$$

(10) $$\text{Recall}=\frac{TP}{TP+FN}$$

(11) $$\text{ Mean Dice=}\frac{2TP}{2TP+FP+FN}$$

Where, True Positive (TP) represents the number of pixels correctly identified as roots by the model. False Positive (FP) represents the number of background pixels incorrectly identified as roots. True Negative (TN) represents the number of pixels correctly identified as the background. False Negative (FN) represents the number of root pixels incorrectly identified as the background.

(12) $$T_{prec}=\frac{\left|S_{P}\cap G\right|}{|S_{P}|+\epsilon }$$

(13) $$T_{sens}=\frac{\left|S_{G}\cap P\right|}{|S_{G}|+\epsilon }$$

(14) $$\text{Centerline-Dice}=\frac{2\times T_{prec}\times T_{sens}}{T_{prec}+T_{sens}+\epsilon }$$

Where, *P* represents the result of predicted segmentation; G represents ground-truth label; *S_p_* represents the skeleton of the predicted mask P;*S_G_* represents skeleton of the ground-truth mask G; ϵ represents a small constant used to avoid division by zero; *T_prec_* denotes the proportion of the predicted skeleton that lies within the ground-truth root region. *T_sens_* denotes the proportion of the ground-truth skeleton that is covered by the predicted root region.

### 2.5 Comparative test

The main purpose of our comparative experiments is to select a well-performing baseline model on the hydroponic maize root dataset. We selected several widely used image segmentation models for comparison, including UNet++ (Zhou et al., 2018), SegNeXt (Guo et al., 2022), SegFormer (Xie et al., 2021), DeepLabV3+ (Chen et al., 2018), and UNet. Considering the rapid development of promptable foundation segmentation models in general image segmentation tasks in recent years, we further introduced SAM2 (Ravi et al., 2024)as a representative model of the SAM series for comparative experiments. In addition, we also attempted to evaluate the root segmentation performance of SAM3 under a zero-shot text-prompt setting. As a new-generation promptable foundation segmentation model, SAM3 (Carion et al., 2025) supports language, exemplar, and visual prompts, and has open-vocabulary object segmentation capability. However, in the hydroponic maize root images used in this study, the root targets are slender, sparse, low-contrast, and highly topologically continuous. In addition, background reflection exists under the hydroponic imaging environment. These factors make it difficult to stably localize the complete root structure using only general text prompts. Therefore, SAM3 was not included in the main quantitative comparison table in this study. To ensure fairness, all supervised segmentation models were trained and tested under the same settings. We evaluated these six models using five metrics: Mean Intersection over Union (mIoU), Foreground Intersection over Union (FG IoU), Recall, Dice Similarity Coefficient and Centerline-Dice. The results are shown in **Table 2**.

**Table 2:** Comparative experiments results.

Model	mIoU/%	FG IoU/%	Recall/%	Mean Dice/%	Centerline-Dice/%
UNet	90.10	80.94	85.82	94.50	95.73
Deeplabv3+	85.32	71.62	82.67	91.48	83.61
Segformer	85.44	71.87	84.57	91.57	84.01
Segnext	85.75	72.60	77.18	84.12	87.85
UNet++	89.17	79.07	85.12	86.94	94.37
SAM2	79.15	64.83	87.69	75.16	79.71

**Figure 4:**
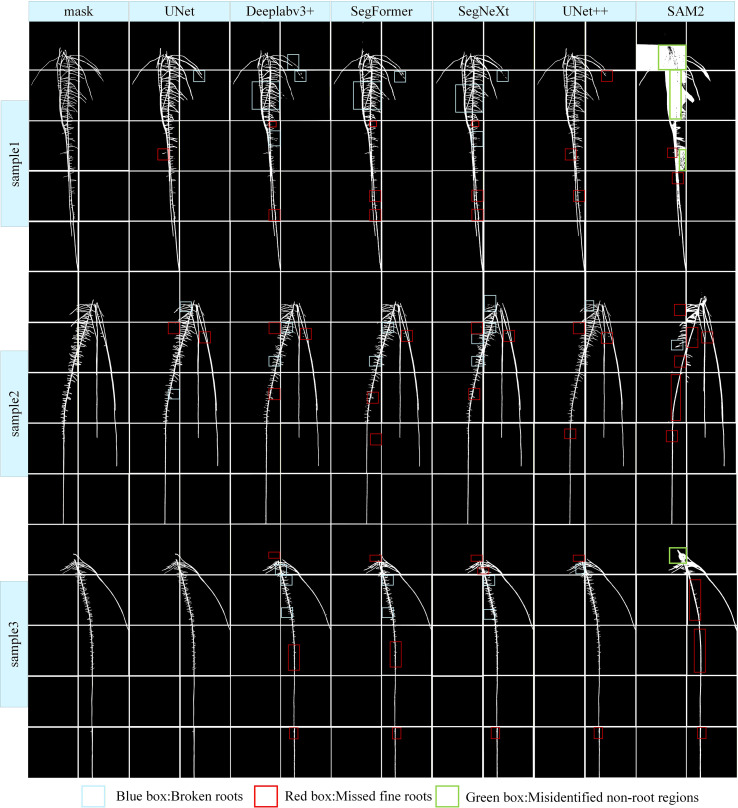
Detection effect diagrams of different semantic segmentation models on the test set.

As shown in **Table 2**, the UNet model achieves the best performance across all evaluation metrics. Its mIoU reaches 90.10%, which is 0.93 percentage points higher than the second-best model, UNet++. Its FG IoU is 80.94%, significantly higher than the other models. This indicates that UNet has better localization and segmentation accuracy for the target root regions. Its Recall and Mean Dice are 85.82% and 94.50%, respectively. This shows that the model performs well in fully capturing the root structure and reducing the missed detection rate. In contrast, UNet achieved the highest Centerline-Dice among the baseline models, indicating better preservation of root continuity and skeleton-level structure. For a more intuitive comparison, we show the root segmentation results of the five models in **Figure 4**. In these images, blue indicates broken roots, red indicates missed fine roots and green indicates areas where the background was incorrectly classified as roots. DeepLabV3+, SegFormer, and SegNeXt show severe cases of broken roots and missed fine roots. UNet++ performs poorly in detecting fine roots. The SAM model showed the poorest segmentation performance. Most fine roots could not be identified, and severe misclassification occurred, with background regions being incorrectly recognized as roots. Overall, the segmentation results of the UNet model show fewer broken roots and missed fine roots. In summary, the UNet model demonstrates the best overall performance in the hydroponic maize root segmentation task, making it an ideal baseline model for subsequent improvements in this study.

### 2.6 Ablation test

To evaluate the impact of different improved modules on the performance of the DB-UNet model, we conducted ablation experiments based on the classic UNet. We gradually integrated a lightweight ViT branch, the Attention Fusion Module (AFM), and a hybrid loss function to construct the final DB-UNet architecture. During training, all other parameters were kept consistent except for the specific modules being tested. The results of the ablation experiments are shown in **Table 3**.

**Table 3:** Ablation test results.

Model	LOSS	mIoU/%	FG IoU/%	Recall/%	Mean Dice/%	Centerline-Dice/%
UNet	Dice loss	90.10	80.94	85.82	94.50	95.73
D-UNet	Dice loss	89.26	79.37	83.30	93.62	94.92
DB-UNet	Dice loss	90.63	82.04	92.95	94.69	96.34
DB-UNet	SL loss	91.02	82.78	89.36	95.02	97.72

**Figure 5:**
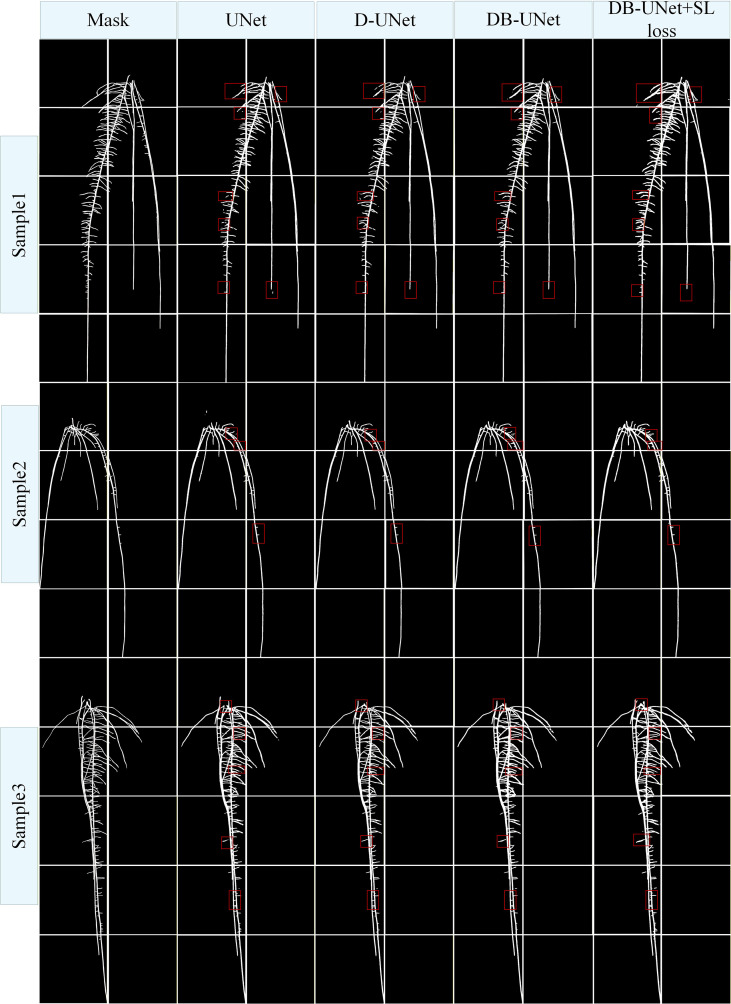
Effect detection diagram of the improved model on the test set.

After incorporating the lightweight ViT branch, performance decreased. Specifically, mIoU dropped from 90.1% to 89.26%, FG IoU from 80.94% to 79.37%, Recall from 85.52% to 83.30%, Mean Dice from 94.50% to 93.62%, Centerline-Dice from 95.73% to 94.92%. This decline occurred because CNNs extract local detail features rich in texture and edges, whereas ViT extracts global contextual features. A significant semantic gap exists between these two. Simple channel concatenation fails to align these heterogeneous features, leading to information conflict and redundancy in the feature space. This interferes with the network’s ability to judge small root pixels. To address these issues, we further introduced the AFM module. As a result, mIoU rose to 90.63%, FG IoU increased to 82.04%, foreground Recall rose to 92.95%, Mean Dice reached 94.69% and Centerline-Dice reached 96.34%. This indicates that dynamic weight allocation effectively preserves the structural information extracted by the Transformer while filtering out redundant noise in low-level features. And the AFM allows the model to rely more on CNN-derived local details in root edge and root tip regions, while assigning greater importance to ViT-derived global context when preserving primary-root connectivity and overall root structure. It achieves a complement between local details and global macro information. Therefore, the improvement of DB-UNet is not simply due to increased model complexity, but results from a task-oriented feature fusion strategy designed for the structural characteristics of maize roots. With the further introduction of the hybrid loss function, mIoU increased to 91.02%. Although the Recall decreased slightly compared to the previous experiment, it remained higher than that of the baseline model. Furthermore, the FG IoU reached 82.78%, demonstrating that the model performs better in reducing misjudgments and improving precision. Centerline-Dice reached the highest value among the ablation models. **Figure 5** illustrates the segmentation effects of the model on hydroponic maize roots after adding each module.

**Figure 6:**
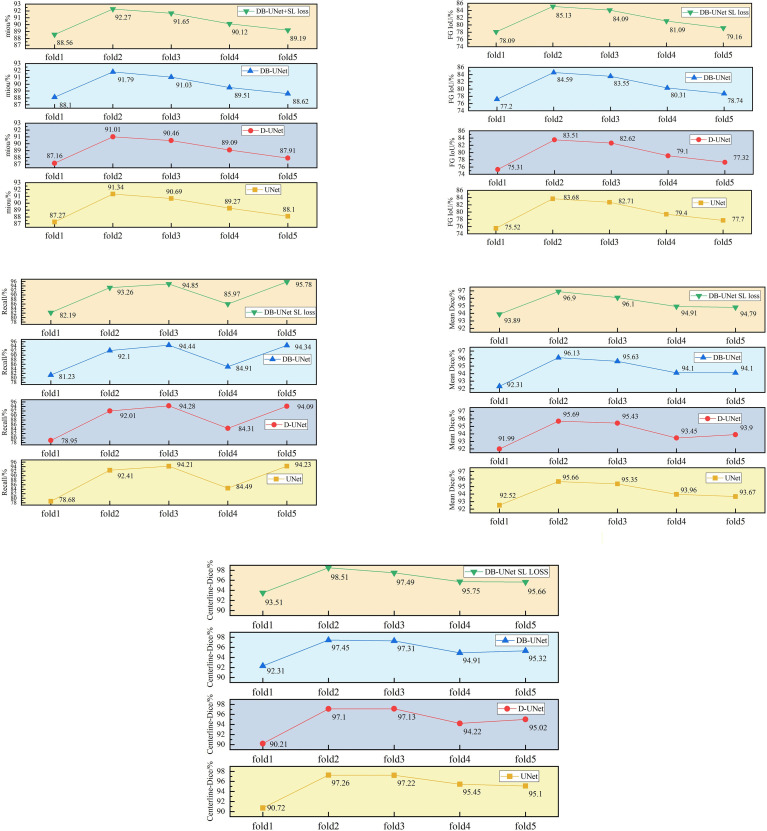
The results of different models under five-fold cross-validation.

In addition, to further evaluate the robustness and generalization ability of the improved DB-UNet model under the condition of a limited number of plant individuals, we conducted plant-level five-fold cross-validation. The 22 maize plants were randomly divided into five non-overlapping subsets. In each round, one subset was used as the test set, while the remaining plants were used for model training and validation. All original images, cropped patches, and corresponding annotation masks derived from the same maize plant were consistently kept within the same fold throughout the experiment. The results showed that DB-UNet maintained stable segmentation performance across different plant-level partitions. The experimental results are presented in **Table 4**, and the detailed results for each fold are shown in **Figure 6**.

**Table 4:** Mean results of each ablation model under five-fold cross-validation.

Model	LOSS	mean mIoU/%	mean FG IoU/%	mean Recall/%	mean Dice/%	mean Centerline-Dice
UNet	Dice loss	89.33	79.80	88.80	94.23	95.15
D-UNet	Dice loss	89.13	79.57	88.72	94.09	94.73
DB-UNet	Dice loss	89.81	80.88	89.40	94.45	95.46
DB-UNet	SL loss	90.36	81.51	90.41	95.32	96.18

## 3 Root phenotyping framework and multi-trait analysis

### 3.1 Establishment of root length measurement benchmark and method development

#### 3.1.1 Establishment of WinRHIZO reference measurements for root length evaluation

To ensure the objectivity, reproducibility, and comparability of root length measurements, the WinRHIZO root image analysis system was selected as the extraction tool in this study. As a key root phenotypic trait reflecting plant growth status, spatial expansion capacity, and water and nutrient acquisition ability, root length has been widely used in crop root phenotyping and growth evaluation, owing to its robustness and ease of quantification in image-based analysis (Alsalem et al., 2021; Delory et al., 2017).

WinRHIZO is one of the most widely adopted automated image analysis systems in plant root phenotyping, capable of extracting total root length and multiple morphological parameters from scanned root images, and has been extensively validated for its stability and reproducibility in a wide range of studies (Bouma et al., 2000; Martin et al., 2022). Compared with manual root tracing, WinRHIZO significantly improves analysis efficiency and reduces human-induced errors, making it particularly suitable for high-throughput root image processing. In addition, compared with general-purpose image processing software (e.g., ImageJ), WinRHIZO incorporates root-specific algorithmic modules designed for automated root length estimation and morphological quantification, thereby improving measurement consistency.

Although recent advances in deep learning–based root analysis methods have shown promising performance, such approaches typically rely on large annotated datasets and model training procedures, and their generalization ability across experimental conditions remains uncertain. Therefore, WinRHIZO is still widely regarded as a standard tool in conventional root image analysis when a consistent evaluation reference is required.

It should be noted that the measurements obtained by WinRHIZO still depend on image quality and preprocessing procedures, including thresholding settings, separation of overlapping root structures, and noise removal. In dense or highly complex root systems, under-segmentation or over-segmentation may occur, potentially introducing systematic bias in root length estimation. Accordingly, the outputs of WinRHIZO in this study are treated as a reference standard rather than an absolute ground truth.

All root images were scanned at a resolution of 164 DPI. A calibration procedure was performed to determine the spatial resolution as 0.142 mm·pixel^-^¹. Based on this calibration, pixel-based measurements were converted into physical lengths, enabling quantitative estimation of root length. Finally, root length for each sample was automatically calculated using WinRHIZO and used as a unified reference benchmark for subsequent model evaluation.

#### 3.1.2 Root length measurement based on a customized skeleton algorithm

Root length estimation based on skeleton representation has been widely adopted in root phenotyping studies. Although the traditional Zhang–Suen parallel thinning algorithm can achieve single-pixel skeleton extraction for root length estimation (Zhang and Suen, 1984), it exhibits two inherent limitations in practical applications. First, the algorithm is highly sensitive to residual noise and minor adhesions, which often results in the generation of numerous spurious branches, leading to an overestimation of skeleton length. Second, conventional chain code–based methods tend to produce duplicate counting around branching points, further amplifying measurement errors. To overcome these limitations, an improved root length estimation algorithm is proposed. By integrating burr pruning based on breadth-first search (BFS) and adjacency graph traversal based on depth-first search (DFS), the proposed method effectively suppresses noise-induced artifacts and eliminates repeated counting at the structural level.

To enable efficient and quantitative analysis of segmentation results, a root length estimation framework based on skeleton extraction and improved chain code statistics is developed. While preserving the intrinsic topological structure of the root system, this method significantly reduces noise interference and redundant counting, thereby improving both measurement accuracy and computational efficiency. The main steps are described as follows:

##### 3.1.2.1 Image preprocessing and connected component analysis

The segmented root image is modeled as a binary function, where I(p)=255 and I(p)=0 denotes background pixels.

Connected component labeling was first performed based on 8-neighborhood connectivity. Isolated noise was removed using an area threshold $\tau_{area}=50$, retaining only valid root regions. For images containing multiple connected components, further classification was conducted to distinguish the primary root from lateral roots. The largest connected component was identified as the primary root, while the remaining components were grouped as lateral roots, providing a basis for subsequent component-wise measurement.

##### 3.1.2.2 Root skeleton extraction

Skeleton extraction is a critical step for root length estimation, aiming to reduce the root region to a one-pixel-wide representation while preserving its topological structure. In this study, the Zhang–Suen parallel thinning algorithm is applied to iteratively refine the binary image. The algorithm removes boundary pixels through two sub-iterations while maintaining connectivity, progressively shrinking the root region until convergence. The resulting skeleton *S* satisfies the following properties:①Single-pixel width: each skeleton pixel has no more than two foreground neighbors within its 8-neighborhood;②Topology preservation: the skeleton remains homotopically equivalent to the original root structure, preserving all branching information.

This step yields a compact topological representation of the root system, avoiding the coarse-grained errors associated with area-based approaches.

##### 3.1.2.3 Burr pruning

Skeletonization is susceptible to noise, which may introduce short spurious branches (burrs). The skeleton *S* is modeled as an undirected graph G=(V,E), where nodes *V*correspond to skeleton pixels and edges *E* represent 8-neighborhood connectivity. Endpoint nodes (degree=1) and branching nodes (degree≥3) are identified. Starting from each endpoint, the algorithm traces the unique path to the nearest branching node. If the path length is shorter than a predefined threshold, it is classified as a burr and removed. This operation effectively eliminates noise-induced pseudo-branches while preserving genuine fine root structures, thereby preventing overestimation of root length.

##### 3.1.2.4 Improved chain code–based length estimation

Traditional chain code methods tend to produce duplicate counts at branching points. To address this issue, the pruned skeleton is represented as an undirected graph, and depth-first search (DFS) is employed to ensure that each edge is traversed only once.Specifically, traversal is initiated from all endpoints, and each edge is counted uniquely. Diagonal connections are assigned a length of  √2, while horizontal and vertical connections are assigned a length of 1, enabling precise path length accumulation. At branching points, only the current path is accumulated, and each sub-branch is evaluated independently to avoid redundancy.

The final skeleton length *N*skeleton provides an unbiased estimation of the true topological length of the root system, effectively resolving the repeated counting issue inherent in conventional methods.

### 3.2 Comparative evaluation of the proposed skeleton algorithm and the Zhang–Suen method

**Figure 7:**
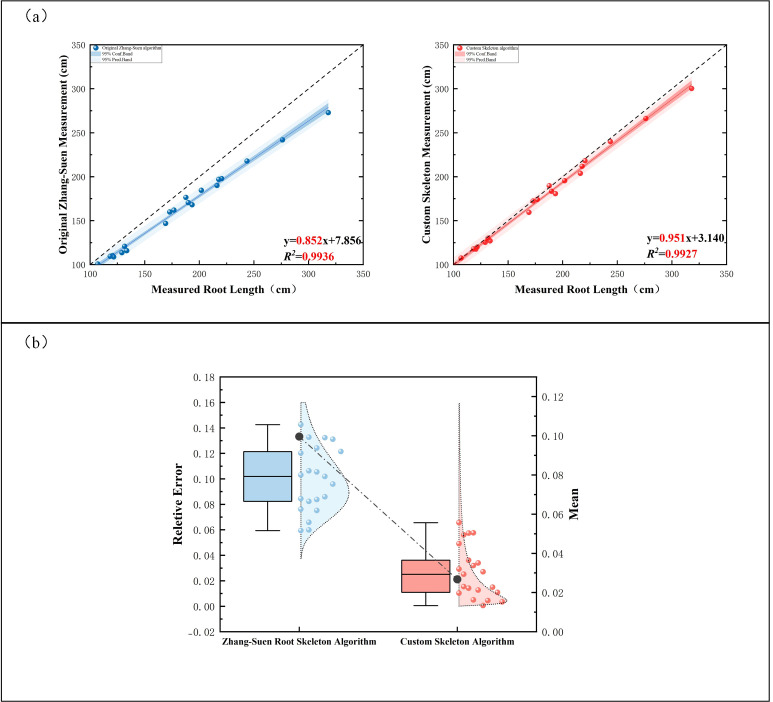
**(a)** Comparison of linear regression analysis between the original Zhang–Suen parallel algorithm and the proposed skeleton algorithm **(b)** Comparison of relative error distributions in root length measurement between the original Zhang–Suen parallel algorithm and the proposed skeleton algorithm.

Based on the segmentation results generated by the optimized DB-UNet model, a comparative evaluation was conducted between the proposed customized skeleton extraction algorithm (incorporating burr pruning and improved chain code statistics) and the conventional Zhang–Suen skeletonization method for root length estimation. The experimental results were benchmarked against ground truth measurements obtained using WinRHIZO. The performance of the two methods was systematically assessed from two perspectives, namely measurement error and result consistency, as presented in **Table 5** and **Figure 7**. 

**Table 5:** Performance comparison of the proposed skeleton algorithm and the Zhang–Suen method for root length measurement.

Image ID	Measured root length by proposed skeleton algorithm (cm)	Measured root ;ength by Zhang–Suen Algorithm (cm)	Ground truth (cm)	Relative error of proposed algorithm	Relative error of Zhang–Suen Algorithm
Image1	125.351	113.598	129.123	2.9212%	12.0234%
Image2	117.473	110.318	120.495	2.5088%	8.4460%
Image3	218.165	197.725	220.443	1.0334%	10.3056%
Image4	239.934	217.778	243.678	1.5365%	10.6288%
Image5	119.845	108.754	121.572	1.4206%	10.5435%
Image6	126.986	115.827	133.540	4.9079%	12.2642%
Image7	300.294	272.754	318.119	5.6032%	14.2604%
Image8	265.177	241.867	276.155	3.6132%	12.4162%
Image9	117.830	109.396	118.417	0.4672%	7.6180%
Image10	129.948	120.785	131.621	1.2711%	8.2327%
Image11	83.134	77.618	83.089	0.0542%	6.5845%
Image12	107.456	100.631	106.983	0.4421%	5.9374%
Image13	174.127	161.964	176.777	1.4991%	8.3795%
Image14	189.723	176.436	187.682	1.0875%	5.9921%
Image15	159.455	146.778	169.160	5.7372%	13.2313%
Image16	180.682	168.018	193.372	6.5625%	13.1115%
Image17	172.294	159.882	172.885	0.3418%	7.5212%
Image18	195.361	184.468	201.809	3.1952%	8.5928%
Image19	183.410	170.510	189.859	3.3967%	10.1912%
Image20	211.863	196.899	217.765	3.2710%	9.5819%
Image21	203.759	189.964	216.222	5.7640%	12.1440%

The quantitative comparison results, as summarized in **Table 5**, clearly demonstrate the superiority of the proposed method. Across the twenty one test samples, the relative error of the improved algorithm was consistently controlled within the range of 1.03% to 5.60%, with an average relative error of only 3.14%. In contrast, the traditional algorithm exhibited substantially higher errors, ranging from 8.45% to 14.26%, with an average of 11.56%, which is approximately 3.68 times greater than that of the proposed method.

Notably, in samples with complex root architectures, the improved algorithm achieved a remarkably low relative error of 1.03%, whereas the traditional method still produced an error as high as 10.31%. This result highlights the strong adaptability and robustness of the proposed method under complex structural conditions.

The error distribution characteristics, as illustrated in **Figure 7b**, further confirm the robustness of the proposed approach. The overall distribution range of the improved algorithm is significantly lower than that of the conventional method. Specifically, the interquartile range (IQR) of the proposed method is 2.87%, markedly smaller than the 3.91% observed for the traditional algorithm, indicating reduced variability and enhanced stability. In addition, the mean error of the proposed method is reduced by 8.5 percentage points compared to the conventional approach, and no extreme outliers are observed. In contrast, the traditional algorithm exhibits evident high-error outliers, suggesting inferior reliability.

Further analysis of consistency with ground truth values reveals that the measurements obtained by the proposed method closely align with the WinRHIZO reference, with no observable systematic bias. In comparison, the traditional algorithm consistently underestimates root length, indicating a pronounced systematic bias (Seethepalli et al., 2021).This discrepancy arises because, although the traditional method generates numerous spurious branches, the resulting positive error is insufficient to compensate for the negative error caused by the absence of effective burr removal and the loss of fine root information during thinning.

By contrast, the proposed algorithm achieves a balance between noise suppression (via burr pruning) and structural fidelity (via non-redundant traversal), effectively removing invalid artifacts while preserving the complete skeleton structure. As a result, it enables an unbiased estimation of root length.

Overall, compared with the traditional Zhang–Suen algorithm, the proposed skeleton extraction method significantly reduces measurement errors caused by spurious branches and duplicate counting. Moreover, it demonstrates strong adaptability across diverse root morphologies in maize seedlings, providing an efficient and reliable computational approach for high-throughput and high-precision root phenotyping.

### 3.3 Comparative analysis of root length estimation across different semantic segmentation models

To evaluate the performance of different semantic segmentation models for root system phenotype extraction in maize at the seedling stage, the segmentation results of six models—DB-UNet, U-Net, U-Net++, SegFormer, DeepLabv3+, and SegNeXt—were used as inputs to an improved custom skeleton extraction algorithm for root length calculation. Root length measurements obtained with the WinRHIZO system were taken as ground truth, and the relative errors of the predicted root lengths were calculated for each model. The same set of 21 images used in the previous section was employed for model comparison in this section.

**Figure 8:**
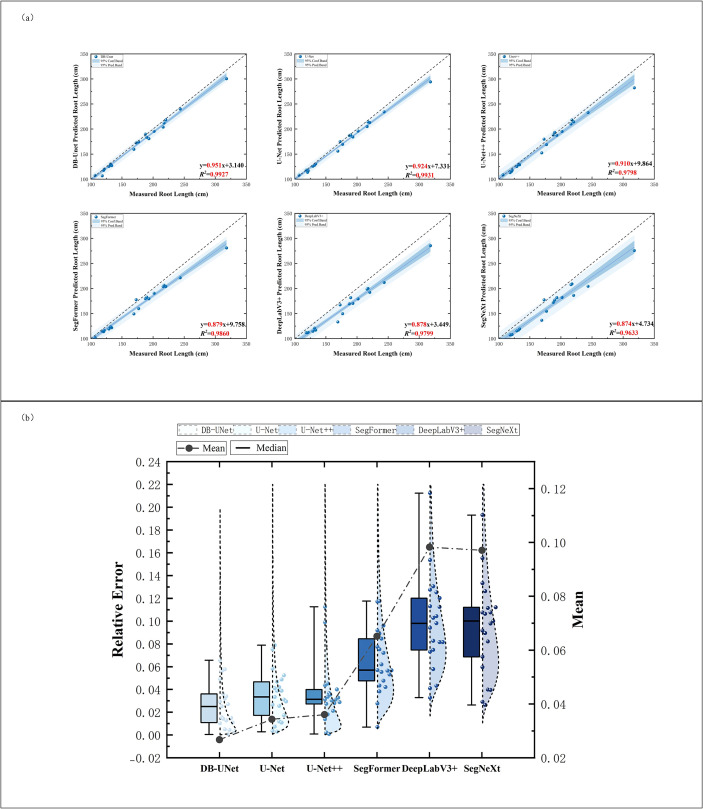
**(a)** Relationship between root projected area calculated from the binary root mask and WinRHIZO reference measurements. **(b)** Relationship between branch point count extracted from the pruned skeleton graph and WinRHIZO reference measurements.

Linear regression analysis was performed on the predicted values of the six models against the ground truth values to assess their consistency, as shown in **Figure 8a**. The results revealed a significant linear correlation between the predicted values and the ground truth for all models. DB-UNet and U-Net exhibited the best fitting performance, indicating that further optimization based on the U-Net architecture is both reasonable and feasible. Moreover, DB-UNet showed the narrowest 95% confidence and prediction intervals, suggesting lower prediction dispersion and higher model stability, further confirming that the DB-UNet model achieves effective optimization. In contrast, although U-Net++ also demonstrated a high degree of fit (R²=0.9798), its slightly wider intervals indicated some variability in prediction. SegFormer, DeepLabv3+, and SegNeXt yielded relatively lower coefficients of determination (R²=0.9869, 0.9799, and 0.9633, respectively), indicating substantially inferior fitting performance and stability.

The distribution characteristics of prediction errors across models are presented in **Figure 8b**, where a combination of boxplot and scatter plot illustrates the relative error distribution for each model, with dashed lines indicating the average error trend. Overall, the DB-UNet model achieved the lowest mean relative error and the smallest interquartile range, reflecting the most concentrated error distribution and the best stability and robustness. U-Net showed a slightly higher mean error but still outperformed the other models. U-Net++ exhibited a marginally higher error level with a relatively concentrated distribution. In contrast, SegFormer, DeepLabv3+, and SegNeXt not only had higher mean errors but also showed greater error dispersion and numerous high-error outliers, indicating insufficient model stability.

Based on the above quantitative analyses, U-Net significantly outperformed U-Net++, SegFormer, DeepLabv3+, and SegNeXt in terms of root length prediction accuracy, fitting stability, and error concentration, confirming that the direction of improvement and optimization based on the U-Net architecture is both reasonable and feasible. On this basis, the proposed DB-UNet model achieved further performance breakthroughs. Compared with the baseline U-Net, DB-UNet exhibited a more concentrated error distribution. These results validate the effectiveness of the designed CNN-ViT dual-branch parallel structure, attention fusion module, and hybrid loss function. They also demonstrate that DB-UNet is better suited to the segmentation requirements of fine roots, small targets, and complex topological structures in hydroponic maize roots, making it the optimal model for high-throughput root system phenotyping at the maize seedling stage.

### 3.4 Multi-trait root phenotyping based on root projected area and branch point count

#### 3.4.1 Rationale for selecting root projected area and branch point count as additional phenotypic traits

Root system architecture (RSA) is a complex morphological and topological trait that cannot be sufficiently characterized by total root length alone (Lobet et al., 2014; Seethepalli et al., 2021).Therefore, to overcome the limitation of single-trait phenotyping and to enhance the multi-trait analytical capability of the proposed phenotyping system, two additional root traits, namely root projected area and branch point count, were further extracted and validated in this study.

Root projected area is a representative two-dimensional morphological trait that reflects the overall spatial coverage of the root system (Seethepalli et al., 2021; Martin et al., 2022). In this study, root projected area was calculated directly from the binary root segmentation mask by counting the number of foreground pixels belonging to the root region. Compared with root length, root projected area is more sensitive to the overall completeness of root segmentation, including missed fine roots and false-positive background regions. Therefore, it can serve as a complementary pixel-level descriptor for evaluating root system size and segmentation completeness.

Branch point count is an important topological trait for characterizing root branching structure and architectural complexity. In this study, branch points were extracted from the pruned root skeleton generated by the proposed customized skeleton-based algorithm (Pagès, 2014; Lobet et al., 2011).

Together, total root length, root projected area, and branch point count describe three different aspects of RSA: one-dimensional root extension, two-dimensional spatial occupation, and topological branching complexity. The extracted root projected area and branch point count were further compared with the corresponding WinRHIZO measurements. In this study, WinRHIZO outputs were used as a consistent reference standard rather than absolute ground truth, because they may still be affected by image quality, thresholding, and root overlap. This multi-trait evaluation strategy provides a more comprehensive assessment of the proposed phenotyping system.

#### 3.4.2 Linear regression fitting between measured and reference values of root area and branching points

**Figure 9:**
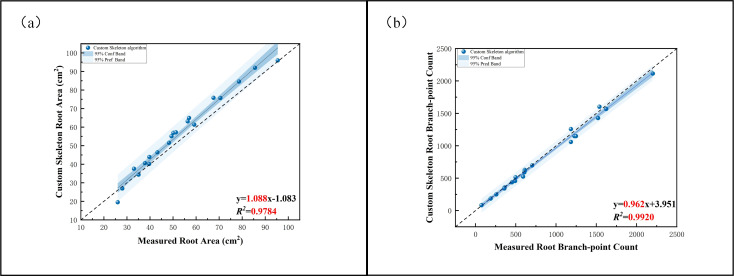
**(a)** Residual diagnostics of root area. Top-left: standardized residuals vs. independent variable; bottom-left: standardized residuals vs. fitted values; top-right: frequency histogram of standardized residuals; bottom-right: normal Q–Q plot of standardized residuals. **(b)** Residual diagnostics of root branch-point. Top-left: standardized residuals vs. independent variable; bottom-left: standardized residuals vs. fitted values; top-right: frequency histogram of standardized residuals; bottom-right: normal Q–Q plot of standardized residuals.

To comprehensively evaluate the ability of the proposed segmentation-based phenotyping framework to quantify root geometric morphology and topological structure, root projected area and branch point count obtained from WinRHIZO were used as reference values. Linear regression analysis was performed to compare the WinRHIZO reference values with the corresponding measurements extracted by the proposed method. Specifically, root projected area was calculated from foreground pixel statistics of the binary root mask, whereas branch point count was extracted from the pruned skeleton graph generated by the customized skeleton-based algorithm. The results are shown in **Figure 9**.

For root area, the regression results (**Figure 9a**) show a linear regression equation of y=1.088x−1.083,with a coefficient of determination R² = 0.9784. The regression slope is close to 1 and the intercept is near 0, indicating a strong linear correlation between the proposed method and the WinRHIZO reference values, with no significant systematic bias. All sample points fall within the 95% confidence interval and prediction interval, further demonstrating the consistency and reliability of the root area measurements.

For root branching point counts, the regression results (**Figure 9b**) yield a regression equation of y = 0.962x + 3.951,with R² = 0.9920. The goodness of fit is higher than that of root area, indicating that the proposed algorithm achieves even better accuracy in capturing topological structural features. The slope slightly below 1 suggests that the algorithm slightly underestimates branching point counts compared to WinRHIZO. This may be attributed to skeletonization errors in regions with root overlaps and intersections; however, the deviation remains small and is within an acceptable range for root phenotyping analysis.

Overall, the high R² values for both root area and branching points indicate that the proposed method not only accurately reconstructs the overall two-dimensional root morphology but also reliably captures topological branching structures. This provides a solid data foundation for subsequent multi-trait evaluation and aboveground–belowground correlation analysis.

#### 3.4.3 Residual analysis of root area and branching point measurements

**Figure 10:**
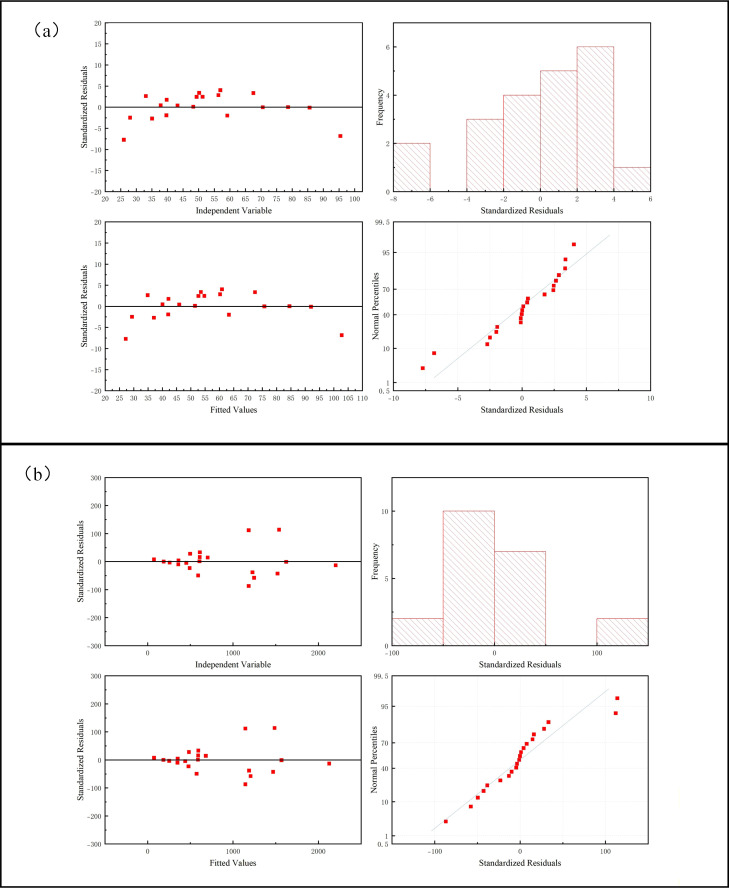
**(a)** Relationship between Centerline-Dice and root length relative error. **(b)** Relationship between mIoU and root projected area relative error.

To further evaluate the error distribution characteristics and method stability, a residual analysis was conducted based on the above linear regression models. The results are shown in **Figure 10**.

For root area measurements, the residual analysis (**Figure 10a**) shows that standardized residuals are evenly distributed around the zero line, without obvious funnel-shaped or curved patterns, indicating no heteroscedasticity or systematic model deviation. The residual histogram is approximately unimodal, and the majority of points in the normal Q–Q plot lie along the diagonal line, suggesting that the residuals approximately follow a normal distribution. The measurement errors are mainly random, with no significant systematic bias.

For root branching point counts, the residual analysis (**Figure 10b**) shows a generally uniform distribution without clear trends. However, a few samples exhibit relatively large residuals. Further analysis indicates that these errors mainly occur in samples with extremely high branching density and severe root overlap, representing local and controllable errors that do not affect overall measurement accuracy. The residual histogram and normal Q–Q plot indicate that most samples follow an approximately normal distribution, demonstrating good overall stability of the method.

In summary, the residual analysis confirms that the proposed skeleton-based algorithm exhibits strong consistency and stability in both root area and branching point measurements. The error levels meet the accuracy requirements for root phenotyping analysis.

### 3.5 Effect of segmentation quality on downstream phenotyping accuracy

To further investigate how segmentation quality affects downstream phenotyping, we analyzed the relationships between segmentation accuracy metrics and phenotypic relative errors. Unlike conventional evaluations that only compare pixel-level segmentation performance, this analysis aimed to determine whether improvements in segmentation quality could be transferred to more reliable root trait extraction.

**Figure 11:**
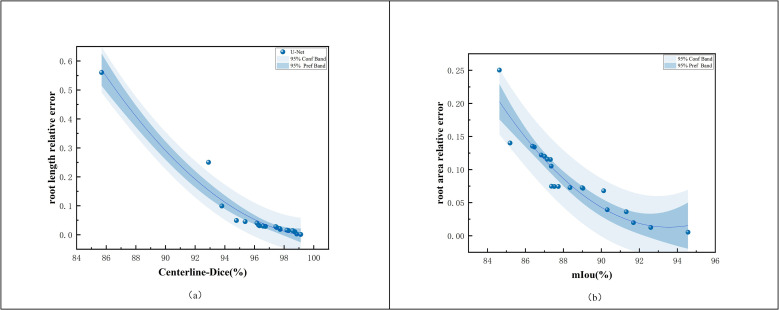
**(a)** Regression analysis of root length predictions by different segmentation models.(b) Distribution of relative errors in root length predictions by different segmentation models.

As shown in **Figures 11a**, Centerline-Dice showed a clear negative relationship with root length relative error. Samples with higher Centerline-Dice values generally exhibited lower root length relative errors, whereas samples with lower Centerline-Dice values showed larger errors in root length estimation. This result indicates that the preservation of root centerline continuity is closely associated with the accuracy of root length estimation. Since root length is calculated based on skeletonized root structures, broken roots, missed lateral roots, and discontinuous skeletons can directly lead to errors in length accumulation. Therefore, Centerline-Dice provides a more phenotyping-relevant evaluation than conventional pixel-level overlap metrics for root length estimation. **Figure 11b** illustrates the relationship between miou and root area relative error. The results show that higher miou was generally associated with lower root area relative error, whereas reduced segmentation consistency led to increased deviation in root area measurement. This is because root projected area is directly derived from the number and spatial distribution of foreground pixels in the segmentation mask. False-positive background regions and missed fine roots can both change the estimated foreground area, thereby increasing the deviation from the reference measurements.

Overall, these results indicate that segmentation quality directly affects the accuracy of downstream root phenotyping. Higher segmentation quality generally leads to lower phenotypic relative errors and more reliable trait measurements. In particular, topology-aware segmentation quality, represented by Centerline-Dice, is closely associated with root length estimation, whereas pixel-level segmentation consistency is more closely related to root area measurement.

### 3.6 Correlation study between shoot and root

There is a close synergistic regulatory relationship between the growth and development of plant shoots and roots (Poorter et al., 2012).This relationship is a core mechanism for plant adaptation to the environment. As the main organ for absorbing water and mineral nutrients, the total root length determines the nutrient supply efficiency to the shoot (Fitter, 2002). Meanwhile, plant height, as a core indicator of shoot growth, reflects the accumulation of photosynthetic products. Therefore, analyzing the correlation features between plant height and root length has important theoretical and practical value.

Based on the crop root phenotyping system, the improved DB-UNet image segmentation model, and a custom root length extraction algorithm, we studied the correlation between the shoot height and root length of maize. The total root length data was obtained by segmenting root images with the improved DB-UNet model and calculating it with an improved chain code statistical algorithm. The plant height data was obtained by locating the top and bottom coordinates of the plants in RGB images. We used a dual-indicator analysis strategy with the Pearson correlation coefficient and the Spearman rank correlation coefficient. The formulas for the Pearson and Spearman correlation coefficients are shown in Equations 15, 16 (Gai, 2013).

(15) $$r_{Pearson}=\frac{\sum_{i=1}^{n}\left(x_{i}-\bar{x}\right)(y_{i}-\bar{y})}{\sqrt{\sum_{i=1}^{n}{\left(x_{i}-\bar{x}\right)}^{2}}\sqrt{\sum_{i=1}^{n}{\left(y_{i}-\bar{y}\right)}^{2}}}$$

Where $x_{i}$ represents the plant height value at the i-th time point, $y_{i}$ denotes the total root length value at the corresponding time point, $\bar{x}$and $\bar{y}$ are the average values of plant height and root length respectively, and n is the number of samples.

(16) $$\rho =1-\frac{6\sum_{i=1}^{n}d_{i}^{2}}{n(n^{2}-1)}$$

Where $d_{i}$​ represents the difference between the plant height grade and root length grade of the i-th sample.

**Figure 12:**
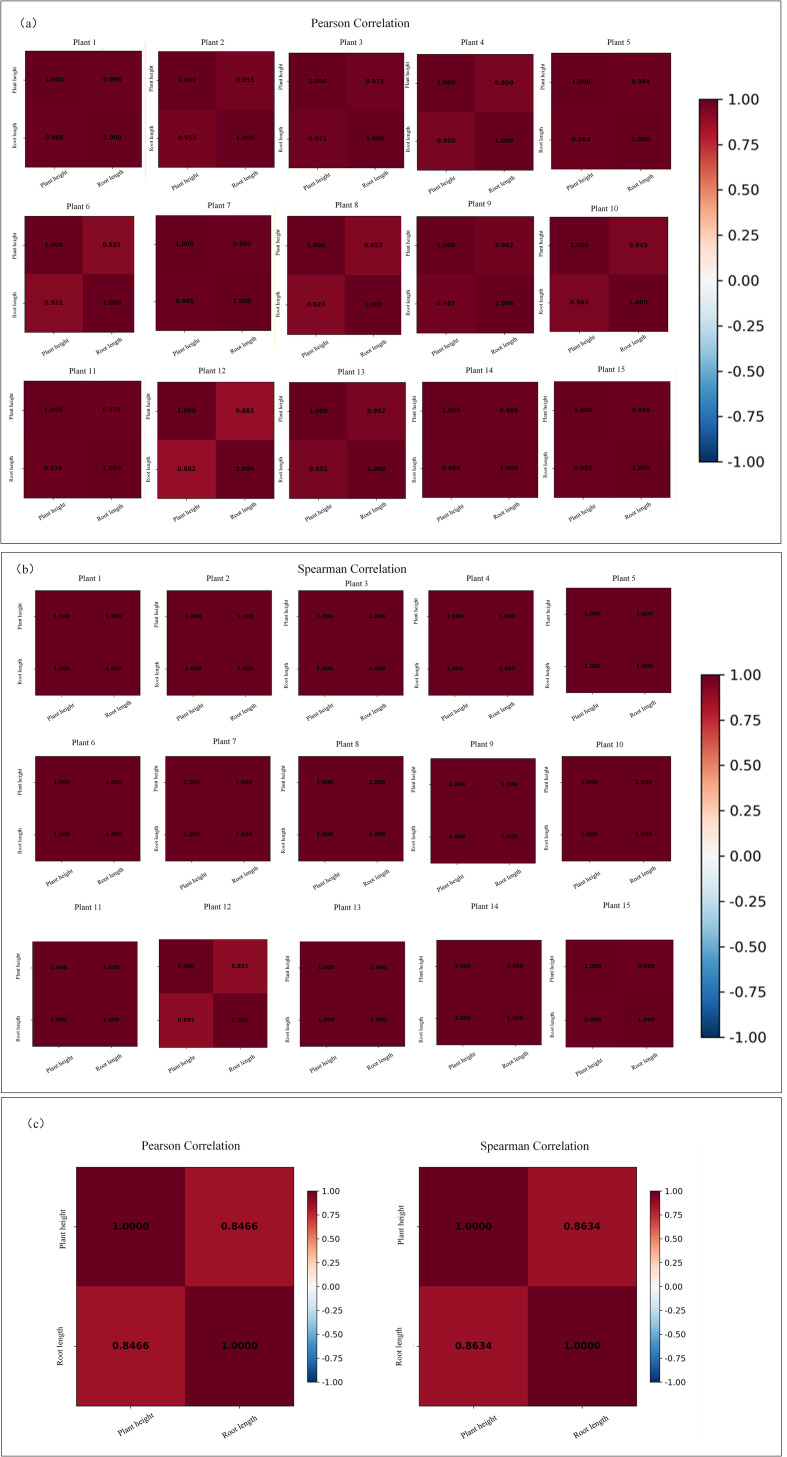
**(a)** Pearson correlation coefficients for 15 individual maize plants **(b)** Spearman correlation coefficients for 15 individual maize plants **(c)** Pearson and Spearman correlation coefficients for the pooled population of 15 maize plants.

**Figure 13:**
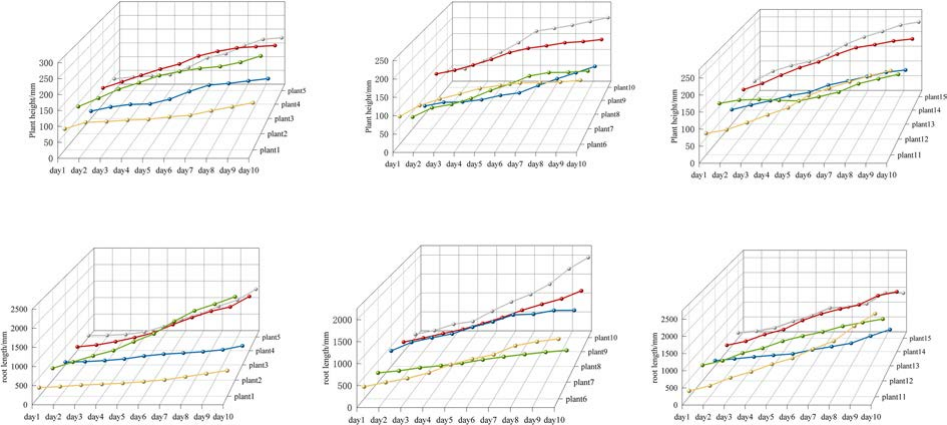
Dynamic changes in aboveground plant height and underground total root length of hydroponic maize during the last 10 days of the growth period.

We selected 15 maize plants and analyzed the correlation between their shoot height and total root length over the last 10 days of their growth cycle. The corresponding Pearson and Spearman correlation coefficient heatmaps are shown in **Figures 12a, b**. The changes in shoot height and total root length over these 10 consecutive days are shown in **Figure 13**. At the individual plant level, the plant height and root length of the 15 maize plants all showed consistent positive correlations. The Pearson correlation coefficients ranged from 0.882 to 0.985. Except for Plant 12 and Plant 15, the Spearman correlation coefficients of the other individual plants reached a perfect positive correlation. This indicates that the growth synchronization between plant height and root length is very strong at the individual scale, with no obvious heterogeneity among individuals. The overall heatmap of the 15 maize plants is shown in **Figure 12c**. The Pearson correlation coefficient was 0.8466, and the Spearman correlation coefficient was 0.8634. Both indicators show a significant and strong positive correlation between plant height and root length. The heatmaps clearly present the correlation features between plant height and root length. The diagonal lines represent the perfect correlation of the variables with themselves. The dark red blocks and high correlation values on the off-diagonals indicate that as plant height increases, total root length shows a significant synchronous growth trend. The Spearman correlation coefficient is slightly higher than the Pearson correlation coefficient. This indicates that the correlation between plant height and root length is not only reflected in a linear relationship, but the consistency of their rank changes is even more significant. This reflects a highly synergistic growth rhythm between the two.

## 4 Conclusion

This study focused on maize root systems at the seedling stage. Traditional root phenotyping methods are destructive and lack *in-situ* monitoring capabilities. Moreover, existing image segmentation models have limited accuracy for fine roots and small targets. To address these issues, we built an *in-situ* imaging dataset for hydroponic maize roots using flat root boxes. We designed a DB-UNet model suitable for segmenting hydroponic maize roots and developed a customized skeleton-based phenotyping framework. We also systematically studied the correlation between maize shoot height and root length. This provides technical support and data reference for high-throughput maize root phenotyping and the study of shoot-root synergistic growth mechanisms. The main conclusions are as follows:

(1) We built a crop root phenotyping system consisting of a crop cultivation module based on transparent flat root boxes and a data acquisition module based on an industrial RGB camera. This system can achieve non-destructive, *in-situ* imaging of maize roots at the seedling stage. It provides high-quality basic data for training and validating root segmentation models, solving the problems of low efficiency and high destructiveness found in traditional root phenotype data collection.
(2) The proposed DB-UNet model is a task-specific optimization for hydroponic maize root phenotyping. By integrating CNN-based local feature extraction, lightweight ViT-based global dependency modeling, attention-based heterogeneous feature fusion, and structural-consistency regularization, DB-UNet more effectively addresses the challenges of fine-root segmentation, including sparse foreground pixels and weak boundaries. The results show that DB-UNet achieved consistent improvements in segmentation accuracy and provided more stable and reliable segmentation inputs for skeleton-based root length estimation and subsequent phenotypic analysis. These findings indicate that the value of the proposed method is not limited to the moderate improvements in mIoU or Dice, but is more importantly reflected in its stable support for downstream root phenotypic trait extraction.
(3) Based on DB-UNet segmentation results, the proposed phenotyping framework integrates pixel-based statistics and customized skeleton analysis to extract three representative root traits: total root length, root projected area, and branch point count. Root projected area was calculated from foreground pixels in the binary root mask, while total root length and branch point count were extracted from the pruned skeleton generated by the customized skeleton-based algorithm. Compared with WinRHIZO reference measurements, the proposed method achieved a mean relative error of 3.14% for root length estimation, which was 8.42 percentage points lower than that of the conventional Zhang–Suen method. In addition, root projected area and branch point count showed strong linear agreement with WinRHIZO reference values. These results indicate that the proposed framework involves root extension, spatial occupation, and branching topology, providing reliable support for high-throughput maize root phenotyping.
(4) segmentation metrics to topology-aware and phenotyping-oriented validation. Centerline-Dice was introduced to evaluate the preservation of root skeleton continuity, while multiple phenotypic traits, including total root length, root projected area, and branch point number, were used to validate the downstream applicability of the proposed method. The analysis of segmentation quality and phenotypic relative errors further demonstrated that improved segmentation quality, especially better topology preservation, leads to more accurate and stable root trait extraction. These findings confirm that the proposed DB-UNet and skeleton-based phenotyping algorithm provide a reliable technical framework for high-throughput and multi-trait maize root phenotyping.
(5) The correlation analysis between maize shoot height and total root length shows that both Pearson and Spearman correlation coefficients indicate a significant and strong positive correlation between the two. Furthermore, the Spearman correlation coefficient is slightly higher than the Pearson correlation coefficient. This shows that the correlation between maize plant height and root length is not only reflected at the linear numerical level, but the consistency of their growth rank changes is even more significant. This reflects a highly synergistic growth rhythm between the shoots and roots of maize at the seedling stage.

Although the proposed DB-UNet model and phenotyping framework achieved relatively strong performance on the maize root dataset, several limitations remain to be addressed.

First, the number of biological individuals used in this study was relatively limited. Although plant-level five-fold cross-validation was conducted to evaluate model stability, all images were derived from 22 maize seedlings. This sample size may not fully capture the morphological variation of root systems across different maize genotypes, developmental stages, or environmental treatments. In future work, we will further expand the dataset by including more biological individuals, multiple maize varieties, and longer growth periods.

Second, the current images were acquired under highly controlled hydroponic conditions using transparent root boxes and a stable imaging environment. Such conditions are beneficial for obtaining high-resolution root images and evaluating fine-root segmentation performance, but they are substantially less complex than soil-based or field root environments. Therefore, future studies will further expand the data acquisition scenarios and construct root image datasets covering different cultivation methods, imaging backgrounds, maize varieties, and developmental stages.

Finally, the current framework does not include explicit RSA reconstruction or graph-based structural modeling of root systems. In this study, branch point count was extracted from the pruned skeleton; however, the framework does not identify root orders, reconstruct parent–child relationships among roots, infer tip-to-source paths, or generate RSML representations. Therefore, the current method is suitable for segmentation-driven morphological trait extraction, but it cannot replace graph-based RSA reconstruction frameworks such as RootNav 2.0 or RootEx 2.0. In future work, we will further integrate keypoint detection, root graph construction, root-order classification, and RSML output, thereby extending the current framework from skeleton-based trait extraction toward explicit RSA reconstruction.

In summary, this study developed an *in situ* image-based phenotyping framework for hydroponic maize seedling roots by integrating DB-UNet semantic segmentation with customized skeleton-based trait extraction. The framework enables the extraction of representative morphological traits, including total root length, projected root area, and branch point count, and provides stable support for segmentation-driven root phenotyping under controlled hydroponic imaging conditions. It has important application value for promoting studies on crop root genetic breeding and stress-resistance improvement. Future research will further expand the dataset, validate the model under more complex growth and imaging conditions, and integrate graph-based root structural modeling methods to extend the framework toward more complete RSA reconstruction.

## Data Availability

The raw data supporting the conclusions of this article will be made available by the authors, without undue reservation.
